# Generative Adversarial Networks for Modeling Bio-Electric Fields in Medicine: A Review of EEG, ECG, EMG, and EOG Applications

**DOI:** 10.3390/bioengineering13010084

**Published:** 2026-01-12

**Authors:** Jiaqi Liang, Yuheng Zhou, Kai Ma, Yifan Jia, Yadan Zhang, Bangcheng Han, Min Xiang

**Affiliations:** 1 Key Laboratory of Ultra-Weak Magnetic Field Measurement Technology, Ministry of Education, School of Instrumentation and Optoelectronic Engineering, Beihang University, Beijing 100191, China; ljq_vip77@buaa.edu.cn (J.L.); zhouyuheng@buaa.edu.cn (Y.Z.); mi8970@buaa.edu.cn (K.M.); jyf@buaa.edu.cn (Y.J.); 2 Zhejiang Provincial Key Laboratory of Ultra-Weak Magnetic-Field Space and Applied Technology, Hangzhou Innovation Institute, Beihang University, Hangzhou 310051, China; 3 State Key Laboratory of Traditional Chinese Medicine Syndrome/National Institute of Extremely-Weak Magnetic Field Infrastructure, Hangzhou 310028, China; zhang-yd20@tsinghua.org.cn; 4 Hefei National Laboratory, Hefei 230088, China

**Keywords:** Generative Adversarial Networks (GANs), bio-electric signals, Electroencephalogram (EEG), Electrocardiogram (ECG), data augmentation, deep learning, medical signal processing

## Abstract

Bio-electric fields—manifested as Electroencephalogram (EEG), Electrocardiogram (ECG), Electromyogram (EMG), and Electrooculogram (EOG)—are fundamental to modern medical diagnostics but often suffer from severe data imbalance, scarcity, and environmental noise. Generative Adversarial Networks (GANs) offer a powerful, nonlinear solution to these modeling hurdles. This review presents a comprehensive survey of GAN methodologies specifically tailored for bio-electric signal processing. We first establish a theoretical foundation by detailing GAN principles, training mechanisms, and critical structural variants, including advancements in loss functions and conditional architectures. Subsequently, the paper extensively analyzes applications ranging from high-fidelity signal synthesis and noise reduction to multi-class classification. Special attention is given to clinical anomaly detection, specifically covering epilepsy, arrhythmia, depression, and sleep apnea. Furthermore, we explore emerging applications such as modal transformation, Brain–Computer Interfaces (BCI), de-identification for privacy, and signal reconstruction. Finally, we critically evaluate the computational trade-offs and stability issues inherent in current models. The study concludes by delineating prospective research avenues, emphasizing the necessity of interdisciplinary synergy to advance personalized medicine and intelligent diagnostic systems.

## 1. Introduction

Bio-electric fields, arising from the aggregate electrical activity of excitable cells, serve as fundamental carriers of physiological information within living organisms. These signals are indispensable for monitoring physiological health, diagnosing pathologies, and elucidating the functional mechanisms of the nervous and muscular systems. In clinical medicine, the most prominent manifestations of these bio-electric activities include the Electrocardiogram (ECG), Electroencephalogram (EEG), Electromyogram (EMG), and Electrooculogram (EOG). Specifically, the ECG captures the electrical potential differences generated during the cardiac cycle, reflecting the depolarization and repolarization of myocardial cells, and remains the gold standard for diagnosing cardiovascular anomalies [1]. The EEG records the fluctuating electrical potentials of the cerebral cortex, providing critical insights into brain function and states such as sleep stages and epilepsy. The EMG detects the electrical potentials produced by muscle fibers during contraction, serving as a key tool for evaluating neuromuscular diseases. Finally, the EOG measures the corneo-retinal standing potential to track eye movements, which is vital for ophthalmological diagnosis and human–computer interaction. Collectively, these bio-electric signals form the cornerstone of modern diagnostic medicine.

Despite their diagnostic value, the analysis of bio-electric fields faces substantial challenges. Raw signals are frequently compromised by environmental noise, motion artifacts, and physiological interference [2]. Furthermore, the development of robust analytical models is hindered by inter-subject variability [3], strict data privacy regulations [4], and the prevalent issue of data imbalance [5], where pathological samples are significantly scarcer than normal controls. Conventional signal processing techniques often struggle to reconcile these limitations. However, the advent of Deep Learning (DL) has introduced novel paradigms for medical data analysis. Among these, Generative Adversarial Networks (GANs), introduced by Ian Goodfellow in 2014, represent a breakthrough in generative modeling. GANs employ a minimax game strategy between a generator and a discriminator to synthesize high-fidelity data samples that mimic the underlying distribution of real data [6]. While initially popularized in computer vision [7], GANs have demonstrated immense potential in the medical domain, offering innovative solutions for data augmentation, denoising, and domain adaptation.

The proliferation of GANs has catalyzed a surge of research and subsequent reviews in medical imaging. For instance, Wang et al. [7] critically analyzed GAN structural variants and loss functions within the broader context of computer vision. Gui et al. [8] provided a comprehensive mathematical formulation of GANs, comparing them with other machine learning paradigms across multimedia applications. Specific to medical imaging modalities, Zhou et al. [9] and Yi et al. [10] surveyed GAN applications in image fusion, reconstruction, and segmentation. Furthermore, specialized reviews have addressed GAN usage in PET [11] and multi-modal imaging including MRI and CT [12].

Compared with previous reviews that predominantly focus on the structural analysis of medical imaging (e.g., MRI, CT), this work emphasizes the unique temporal and spectral characteristics of 1D and 2D bio-electric signals. The contributions of this review are reflected in the following aspects. Initially, we provide a systematic taxonomy of GAN applications across four key electrophysiological modalities: EEG, ECG, EMG, and EOG. Given that the research maturity varies across these fields, we adopt a differentiated analytical approach—offering a detailed synthesis for established domains (ECG/EEG) while discussing exploratory efforts in emerging ones (EMG/EOG). Furthermore, this review seeks to complement existing literature by discussing a task-specific evaluation framework. By associating quantitative metrics with core signal processing tasks—including synthesis, denoising, and classification—we identify potential methodological pitfalls such as subject-wise data leakage and metric-task misalignment, with the aim of assisting in maintaining research rigor. Lastly, through a horizontal comparison of maturity levels and technical hurdles across modalities, we discuss the considerations for transitioning research models toward clinical utility. By focusing on both methodological details and the potential for cross-modal integration, this review aims to provide a useful reference for the development of GAN-based electrophysiological analysis.

**Figure 1:**
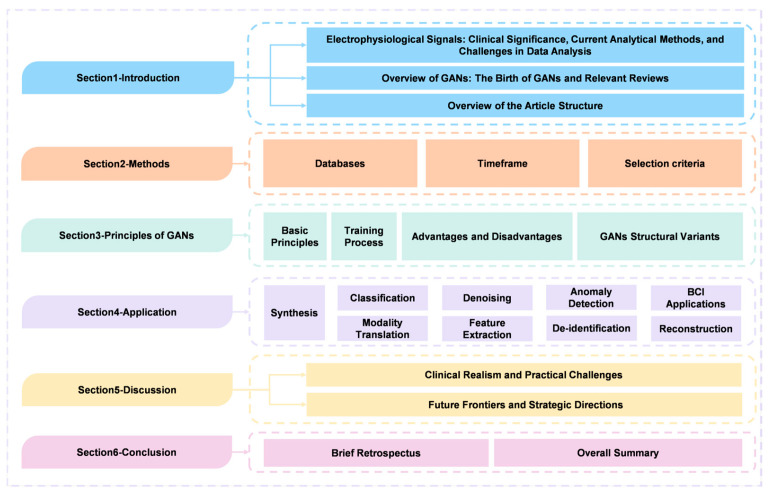
An overview of the structure.

The overview of the structure of this paper is depicted in Figure 1. The structure of this paper is organized as follows: Section 2 outlines the methods employed for literature selection. Section 3 establishes the theoretical foundation, detailing the training mechanisms of GANs and examining structural variants designed to handle signal complexity. Section 4 presents an extensive review of applications, categorizing methodologies into synthesis, classification, denoising, and anomaly detection, while integrating evaluation guidance for each task. Section 5 critically evaluates clinical realism and practical challenges, summarizes the comparative landscape across modalities, and outlines future strategic directions. Finally, Section 6 concludes the paper with a brief retrospect and an overall summary of the field.

## 2. Methods

The literature search for this review was systematically conducted across major academic databases, including PubMed, Web of Science, IEEE Xplore, and Google Scholar. The timeframe for the included studies spanned from 2018 to 2025 to ensure the incorporation of the most recent advancements in the field. The selection followed specific inclusion criteria: First, the study must utilize GANs as a core methodology for electrophysiological signal processing. The literature search was conducted using combinations of keywords such as “GAN” AND “EEG”, “Generative Adversarial Networks” AND “ECG”, as well as similar terms for EMG and EOG. The research focus must involve the generation or analysis of these electrophysiological signals. All included studies must be peer-reviewed and published in English. Regarding data sources, studies based on publicly available datasets were given priority, as these datasets offer higher transparency and reproducibility, and typically have sufficient sample sizes and diversity. For certain specific tasks where public datasets were lacking, studies using proprietary datasets were also considered. We also favored studies that introduced innovative GAN models or optimization methods to address specific challenges in electrophysiological signal analysis. Exclusion criteria included studies unrelated to the application of GANs in electrophysiological signal processing, those that did not propose innovative models or optimization methods, and studies with unreliable data sources or insufficiently rigorous experimental design.

## 3. Overview of GANs

### 3.1. Basic Principles of GANs

GANs are unique neural networks based on generative model framework [13,14,15,16]. Generative modeling is an unsupervised learning process in machine learning that reveals patterns and contextual features in input data to generate new samples with similar characteristics to the original training data set [17]. Typical GANs consist of two main components: the Generator (G) and the Discriminator (D). Inspired by the zero-sum game in game theory, GANs regard the generation problem as a confrontational game between G and D [18]. They achieve data that meets the characteristics of the generation target through confrontational training between G and D [19,20].

In the initial study of GANs, authors used a vivid metaphor to illustrate the contentious relationship between G and D, comparing the training process to a game between a counterfeiter and the police [6,21]. G is a criminal who creates counterfeit money to deceive the police system. And D is police whose goal is to detect counterfeit banknotes. Counterfeiters are working around the clock to update their counterfeiting techniques, and the police are constantly improving their ability to identify authenticity. The two are competing against each other, making the counterfeit banknotes produced more and more realistic. The game between the two continuously promotes both parties’ progress and optimization, revealing the essence of GANs training.

**Figure 2:**
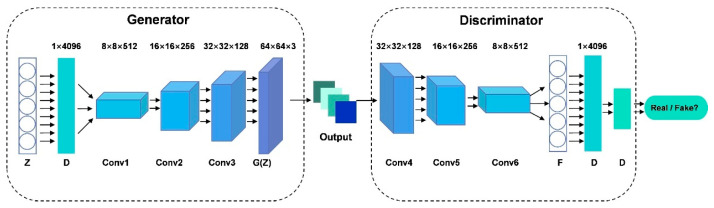
DC-GAN topology example. The generator is connected from a latent space (Z) to a dense layer (D) through progressively upsampled convolution operations to produce a 64 × 64 synthetic data sample. D applies multiple convolutional layers to real or synthetic sample to extract important features. After these features are flattened (F), they are passed to a densely connected network (D) to determine whether the input sample is real or fake.

In general image tasks, typical GANs structure are Deep Convolutional GANs (DC-GANs) [22,23]. They use Convolutional Neural Networks (CNN) as G and D of GANs, replacing the multi-layer perceptron used when Goodfellow et al. first proposed GANs [24]. The specific structure of DC-GANs is shown in Figure 2. Specifically, in DC-GANs, G, as a component of GANs, adopts a neural network model. It takes random noise or latent variables as input and gradually generates synthetic data samples similar to the feature distribution of real data through a series of inverse mapping and transformation operations. The goal of G is to generate realistic samples that are difficult for D to distinguish. Meanwhile, D serves as a binary classifier, and its structure is also a neural network. Its task is to classify the input samples as real or generated data. D receives real data and generated data from G as samples and outputs a probability indicating the authenticity of the sample. During training, D gradually learns to distinguish the feature differences between real and generated data, allowing it to accurately identify the difference between them [25].

From mathematical principles, the core objective of GANs is to train the generator to map a random noise vector z to the distribution of real data x. This learning process is considered successful when the distribution of the generated samples, denoted G(z), becomes as close as possible to the true data distribution of x. For a given real data sample $x\;\sim \;p_{data}(x)$, the system hopes that $G(z)\;\sim \;p_{g}(x)$ approximates $p_{data}(x)$. The discriminator attempts to distinguish between real samples x and generated samples G(z) and outputs the probabilities D(x) and D(G(z)) that they originate from the true data distribution. The adversarial training of G and D can be regarded as a minimum-maximum game process. D hopes to maximize the probability of correctly judging real and generated samples. *G* tries to minimize the probability that D determines G(z) as a false sample. The entire game process can be expressed as an optimization objective:

(1) $$\min_{G}\max_{D}V(D,G)=E_{x\sim p_{data}(x)}[\log D(x)]+E_{z\sim p_{Z}(z)}[\log(1-D(G(z)))]$$

*G* constantly forces D to find it difficult to determine that the samples generated by G are false by reducing V(D,G) in (1). D improves its ability to distinguish between true and false by continuously increasing V(D,G). Through continuous iterative adversarial training, the sample distribution $p_{g}(x)$ generated by G becomes closer and closer to the real data distribution $p_{data}(x)$, and D becomes more and more difficult to judge the authenticity. Finally, G can learn the mapping function that most closely approximates the real data distribution. When the Nash equilibrium point is reached, G can be considered to have learned real data distribution.

### 3.2. GANs Training Process

GANs training is an iterative game process, which can be summarized into the following steps:

1. Initialize the parameters of the G and D.

2. Randomly select a batch of x from the real sample data set.

3. Randomly sample a batch of z from the latent variable space.

4. Input z into G and obtain a batch of generated fake samples G(z).

5. Mix x and G(z) and input them into D.

6. Train D once to maximize the probability of distinguishing x from G(z).

7. Fix *D*’s parameters and train G once based on D’s judgment to minimize the probability of D judging false samples.

8. Repeat steps 2–7 to gradually improve the quality of real samples generated by G and the ability of D to distinguish real samples from fake ones.

9. After reaching the preset number of training cycles or model effect, output the final trained G.

**Figure 3:**
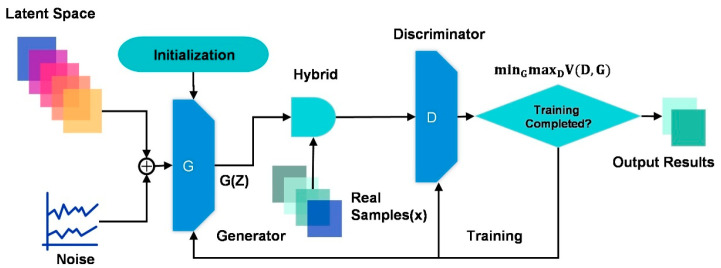
Schematic diagram of GAN training process.

The GANs training process’s basic steps and iterative ideas are as above. Figure 3 illustrates the training process in a visual manner. Note that many variants of GANs currently have different network structures and training process optimizations.

### 3.3. Advantages and Disadvantages of GANs

GANs are important types of current deep generative model. Based on their methodological architecture of adversarial training, GANs exhibit many advantages in electrophysiological data processing tasks:

1. GANs have excellent computing speed in signal processing and can generate new samples efficiently and in parallel. Compared with Boltzmann machines, GANs’ model training process do not require approximate reasoning, so that their learning speed is faster [26]. Compared with pixel-level image generation models such as PixelRNN and PixelCNN, GANs adopt an end-to-end regression method and can generate samples in parallel, making it faster and more efficient in electrophysiological signal processing tasks [27].

2. GANs can approximate the true distribution of data and generate high-quality new samples. This makes GANs outstanding in tasks such as electrophysiological signal analysis, image generation, and text generation [28]. Compared with the famous variational autoencoder (VAE) in deep generative models, the signals generated by GANs are more realistic and natural [29].

3. GANs with flexibility and universality features can handle different types and formats of input data and construct various types of generative models. Therefore, GANs can be applied to different types of electrophysiological signals, such as EEG or magnetoencephalography (MEG) [30]. GANs can also be extended to different generation tasks such as text, image, and audio, and show good performance in different generation tasks. This feature, which does not require the form of input data, makes it more applicable for electrophysiological signal processing [31].

4. GANs adopt an adversarial training mechanism and can learn complex high-dimensional data distribution patterns [32]. The distribution of real data is often complex, with multiple different categories or styles of patterns. Electrophysiological signals also often contain multiple complex patterns, such as different frequency components and spatial patterns. The adversarial training of GANs enables G to capture multiple high-dimensional patterns in data, improving the modeling and sampling capabilities of the complex distribution of electrophysiological signals [4].

5. GANs are compatible with other deep learning models to form a more powerful generation system. For example, GANs can be combined with a VAE for semi-supervised learning, strengthening the coding and expression capabilities of signals and improving model stability. GANs can also be combined with the policy gradient method in reinforcement learning to realize the agent task of modeling the environment and assist policy learning. The combination of these methods provides a more comprehensive solution for electrophysiological signal analysis [33].

Although GANs have many advantages in data generation, as an emerging deep generation model, they still have problems, mainly in the following points:

1. Difficulty in optimizing non-convex functions and achieving convergence and Nash equilibrium. The objective function of GANs is non-convex, which makes it difficult to achieve convergence and reach the global optimal Nash equilibrium state. During training, achieving a balance of synchronous updates between G and D is also difficult. This causes GANs training results to oscillate and become unstable [34].

2. Mode collapse. When a G can successfully generate realistic samples under certain parameters, its learning capabilities and diversity generation capabilities decrease. The samples generated at this time will cluster on several data distribution patterns and lack diversity [35].

3. Gradient vanishing. With iterations, D gradually increases its ability to distinguish real samples from generated ones [36]. In the later stage, D can easily judge the artifacts of the generated samples, causing the gradient signal received by G to disappear. This will cause G unable to continue learning effectively about the real sample distribution, causing gradient vanishing [37].

4. Lack of evaluation metrics. It is theoretically difficult to analyze the convergence of GANs. The assessment of whether GANs converge mainly relies on the evaluation index of the quality of the generated samples. There is a certain degree of subjectivity in sample evaluation, and the effects of different evaluation methods are also different. Therefore, it is difficult to objectively and quantitatively evaluate the convergence of GANs [38].

5. Poor interpretability. As a completely data-driven black box model, GANs have poor interpretability, making its internal representation difficult to understand. This limits model debugging and diagnostics [39].

6. Difficulties in the calculation. Most GANs variants have the problem of long training times and require large-scale computing resource support. Such characteristics are not conducive to rapid iteration [40].

7. Insufficient theoretical research. The adjustment of model parameters requires a great deal of experience, which hinders the application and promotion of GANs [41].

It can be seen that compared with conventional deep generation models, GANs’ framework mechanism based on adversarial training shows many unique advantages, such as high-quality generated samples and the ability to capture the complex distribution of data. This makes GANs particularly suitable for sample generation tasks. However, classic GANs also have some problems, such as poor training stability, and mode collapse. These shortcomings of GANs have also inspired researchers to further transform the structure of GANs, producing many variants of GANs. These variants innovate GANs’ framework so that it can better adapt to different specific problems, thereby leveraging GANs’ unique generative capabilities in various instances [12]. Researchers are also continuing to explore the theory of GANs to find better ways to control and improve the GANs training process.

### 3.4. GANs Structural Variants

The rapid evolution of GANs has led to a proliferation of structural variants, each addressing specific limitations of the original framework. This section examines key developments in GANs architecture, categorized into three primary areas: loss function modifications, conditional generation enhancements, and neural network structural innovations.

**Figure 4:**
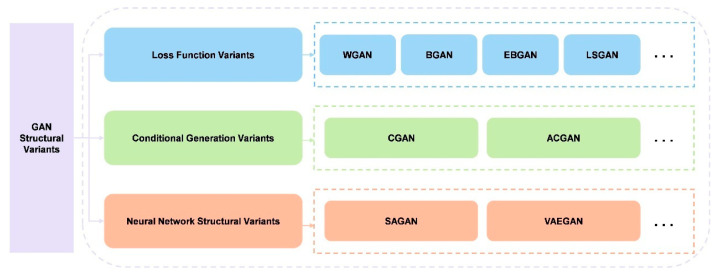
Taxonomy of representative GAN structural variants.

As shown in Figure 4, we propose a taxonomy of representative GANs structural variants, which aims to provide an overview of the field’s progression. This categorization, while not exhaustive, offers a framework for understanding the diverse approaches to GANs development. These variants represent significant strides in improving GANs stability, performance, and applicability across diverse domains. By exploring these structural adaptations, we gain insight into the ongoing efforts to refine and extend the capabilities of adversarial generative models.

#### 3.4.1. Loss Function Variants

The emergence of the Wasserstein Generative Adversarial Networks (WGANs) [42] marks a big step in GANs. By introducing Wasserstein distance as the loss function, WGANs fundamentally solved the problems of training instability and mode collapse faced by the original GANs. Compared with traditional Jensen–Shannon divergence or Kullback–Leibler divergence, WGANs created smoother gradients by minimizing the Wasserstein distance between the generated distribution and the real distribution, effectively improving the training stability of G and D. This improvement aims to bring a more reliable training process to GANs, allowing them to more consistently generate high-quality samples.

At the same time, the introduction of the Boundary-Seeking Generative Adversarial Networks (BGANs) [43] has made innovations in the design of loss functions. BGANs attempted to find identifiable boundaries in G output space by introducing additional boundary functions. The goal of this unique design is to constrain G’s output to maintain smoothness within the input space, thereby significantly improving the consistency of generated samples. BGANs were proposed to make up for the shortcomings of sharp sample boundaries generated by traditional GANs and effectively avoid the problem of mode collapse.

Energy-Based GANs (EBGANs) [44] made contributions based on its theoretical foundation. EBGANs strived to learn the energy distribution of data through energy-based training objectives. G is trained to generate low-energy samples, while D’s task is to ensure that the real samples have lower energy than the generated samples. Compared with the original GANs, which mainly focuses on the limitations of the sample probability distribution, EBGANs emphasizes modeling the energy distribution of the data, providing greater flexibility for the model. This variant is proposed to improve the model’s ability to model complex data distribution and bring richer expressive capabilities to generation tasks.

Least Squares Generative Adversarial Networks (LS-GANs) [45] aimed at a more stable training process. By introducing the least squares loss into GANs, LSGANs replace the binary cross-entropy loss in the original GANs. The advantage of adjusting this loss function is to produce a smoother learning curve, which effectively alleviates the occurrence of the mode collapse problem. By fine-tuning the losses of G and D, LSGANs achieve more reliable and consistent training, helping to ensure the stability of the generation process.

The formulas for the loss function variants discussed in this section are listed in Table 1 To sum up, the introduction of these loss function variants of GANs has solved a series of problems in GANs training from the theoretical and design levels. Each variant has achieved significant improvements in their respective fields, providing a solid foundation for the development of GANs.

#### 3.4.2. Conditional Generation Variants

Conditional GANs (CGANs) [46] introduced conditional information into the development of GANs. The core improvement is that G no longer just generates samples through latent vectors, but introduces additional conditional information, such as category labels. The integration of this condition information allows G to more accurately generate samples that match the given conditions. In CGANs, G and D accept input with conditional information, thereby achieving more refined control over the attributes of the generated samples. The goal of this improvement is to overcome ignoring additional condition information in the original generation process and provide more controllability and personalization for the generation task.

Auxiliary Classifier GANs (ACGANs) [47], as an extension of CGANs, introduced the concept of auxiliary classifiers to further improve G’s ability to generate samples with specified categories. By embedding the classifier in D, ACGANs achieve simultaneous authenticity and category discrimination of generated samples. This variant performs well in combined generation and classification tasks, adding more control and diversity to generated samples. The improvement goal of ACGANs is to strengthen G’s control over generating samples of specific categories, especially in multi-category tasks, to make up for the relative shortcomings of the original GANs in this regard.

The emergence of these two conditional generation variants not only adds sensitivity to conditions to G but also injects richer semantic information into the generation task. The design philosophy of CGANs and ACGANs has made significant progress in overcoming the limitations of original GANs on conditional generation, providing a more flexible and personalized direction for generative models. Through innovative applications of conditional introduction, these two variants demonstrate the powerful potential of GANs in achieving targeted generation tasks.

#### 3.4.3. Neural Network Structural Variants

Self-Attention GANs (SAGANs) [48] introduced a self-attention mechanism to effectively capture long-distance dependencies in input data. The self-attention mechanism enables G to pay more attention to global contextual information when generating high-dimensional data by weighted aggregation of different parts of the input, thereby making up for the limitations of the original GANs in processing long-range temporal sequences or long-distance correlations. Specifically, the self-attention mechanism fuses information at different locations by performing a weighted summation of the input feature maps, thereby improving G’s understanding of the intrinsic structure of the input data. Such improvements not only greatly improve the quality of generated samples but also provide a powerful mechanism for long-range correlation modeling of GANs.

The design of Variational Autoencoder GANs (VAE-GANs) [49] combined the ideas of VAE and GANs to build a more comprehensive, stable, and efficient generative adversarial network. VAEGANs achieves more stable training and better signal generation effects by comprehensively utilizing VAE’s latent space representation and GANs’ generation capabilities. Not only that, VAEGANs also learns more meaningful latent representations while generating samples, further improving the ability to model data distribution. The introduction of this neural network structural variant aims to give full play to the advantages of VAE and GANs to promote more comprehensive and targeted signal generation and latent space learning. These two models are classified as neural network structural variants because their innovations are not only reflected in the adjustment of the loss function or training process but also deeply affect the underlying structure of GANs. SAGANs’ self-attention mechanism and VAEGANs’ design concepts that combine VAE and GANs have both innovated the internal architecture of the neural network. These variants not only improve the modeling capabilities of long-distance dependencies and latent spaces, but also bring a qualitative leap to the stability, effectiveness, and expressiveness of generative models. In general, these neural network structural variants occupy an important position in the evolution of GANs and contribute useful experiences and lessons to the development of deep learning.

In conclusion, the structural variants of GANs discussed in this section exemplify the dynamic nature of research in generative modeling. From reimagined loss functions to sophisticated conditional generation techniques and innovative neural architectures, each variant contributes to addressing the inherent challenges of the GANs framework. These advancements not only enhance the stability and quality of generated samples but also expand the applicability of GANs to more complex and nuanced tasks.

**Figure 5:**
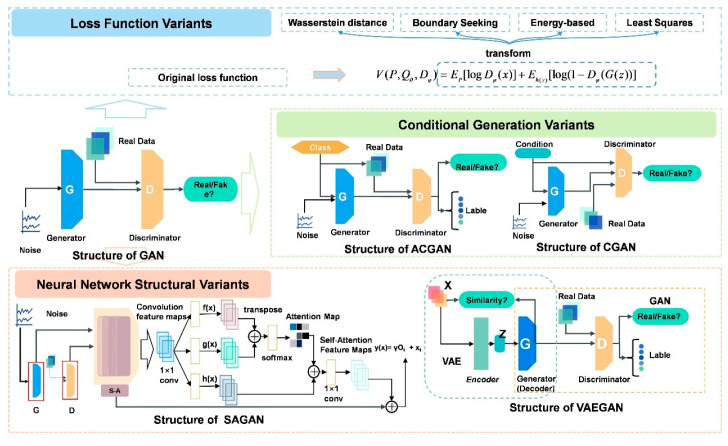
Network structures of representative GAN variants.

Figure 5 provides a visual representation of the network structures for representative GANs variants such as CGANs, ACGANs, SAGANs, and VAEGANs, highlighting the diversity and sophistication of these architectural innovations. As the field continues to evolve, these variants serve as stepping stones, inspiring further innovations and pushing the boundaries of what is possible in generative AI. The ongoing exploration of GANs architectures underscores the potential for continued breakthroughs in this rapidly advancing area of machine learning.

## 4. Applications

In this section, the five main applications of GANs in electrophysiological signal analysis are summarized:

1. Signal synthesis. In medical data, the problem of class imbalance often causes the model prediction results to be biased towards the class with more samples, resulting in low prediction accuracy. GANs can synthesize scarce electrophysiological signal samples to increase the diversity of training data, alleviate the imbalance problem, and thus improve the generalization ability and accuracy of the model.

2. Signal classification. Classification aims to predict the category of the given input data. For example, given an ECG signal, a classification model might predict whether the signal is normal or indicates heart disease. In this case, the model usually already knows all possible categories during the training phase.

3. Denoising. Electrophysiological signals are often interfered with by various noises, for example, motion noise in EMG. GANs can learn noise patterns and generate clean signals without noise, improving signal quality and accuracy of classification.

4. Anomaly detection: Anomaly detection is a critical task in electrophysiological signal processing, aiming to identify signals significantly different from normal patterns that may indicate potential diseases or other important information. GANs can learn the distribution and characteristics of normal electrophysiological signals to detect abnormal ones.

5. Modal conversion: GANs can generate virtual data between modalities in applying multimodal signal conversion and fusion. These data can reflect the complex correlation and common characteristics between multimodal signals.

In addition, the applications of GANs in electrophysiological signal feature extraction, feature identification, signal filtering, and reconstruction are discussed in this chapter. Although relatively little literature is involved, it still has high academic and practical value.

**Figure 6:**
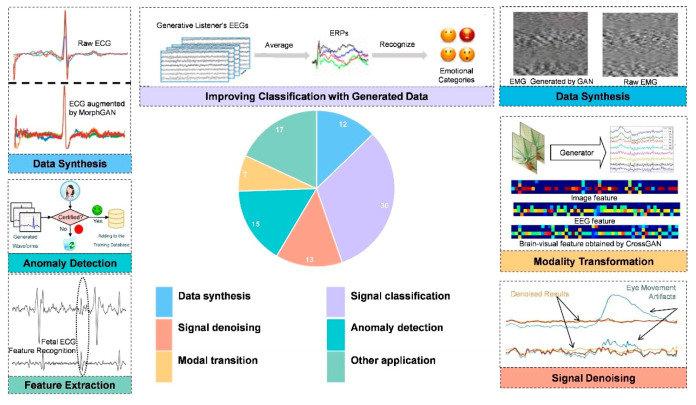
Overview of GAN applications in electrophysiological signal analysis. The pie chart illustrates the distribution of papers and visual examples of GAN functionality across different applications. Specific applications are highlighted as follows: data augmentation using MorphGAN for ECG signals (Reproduced from [50], with permission from IEEE, 2022); generative listener’s EEG for recognizing emotional categories (Reproduced from [51], with permission from IEEE, 2024); GAN-generated EMG data (Reproduced from [52], with permission from Frontiers Media SA, 2022); anomaly diagnosis (Reproduced from [53], with permission from Springer Nature, 2023); extracting fetal ECG (Reproduced from [54], with permission from IOP Publishing, 2021); denoising of signals to remove eye movement artifacts (Reproduced from [55], with permission from IEEE, 2022) and cross-modal transforming (Reproduced from [56], with permission from Elsevier, 2024).

In summary, Figure 6 provides a visual overview of the diverse applications of GANs in electrophysiological signal analysis, with careful consideration given to both application categories and the various electrophysiological signal modalities discussed in this chapter.

### 4.1. Signal Synthesis

In the research and application of electrophysiological signals, obtaining sufficient, high-quality and representative real data is the core challenge [57]. The collection of signals in specific disease states, rare physiological reactions, or under strictly controlled experimental conditions is particularly challenging, resulting in small data sets with insufficient diversity. This scarcity of data severely restricts the training effect and generalization ability of machine learning models. Meanwhile, true physiological signals often contain sensitive personal health information. The use of true physiological signals is subject to strict privacy regulations, which poses a significant privacy dilemma. Traditional data augmentation methods, such as adding noise, time-domain transformation, and resampling, have limited effectiveness when dealing with complex time-series signals and are unable to generate new samples that conform to the actual physiological feature distribution [58].

GANs offer a highly promising solution to address these challenges. In the field of electrophysiological signals, GANs can generate synthetic data that is highly similar to the real signals in terms of statistical characteristics and time–frequency features by their powerful generation capability. This synthetic data significantly alleviates the problem of data scarcity but also provides abundant materials for model training. And the synthetic data does not relate to any specific individual, which can effectively avoid privacy risks. Although there are still challenges in accurately capturing the subtle features of signals and ensuring the diversity of the generated results, GANs have become indispensable innovative tools for advancing electrophysiological signal research in situations with limited data [59].

#### 4.1.1. Synthesis of ECG

**Figure 7:**
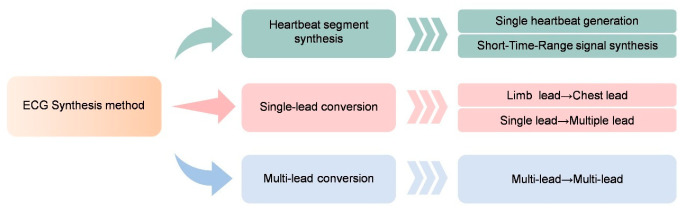
Main research directions of ECG synthesis.

ECG signals synthesis proves invaluable for medical diagnosis, algorithm training, and safeguarding patient privacy. The traditional methods that rely on mathematical modeling (such as dynamic differential equations or piecewise curves) require manual adjustment of parameters to simulate specific pathological features (such as left bundle branch block). This process is complex and difficult to generate diverse signals. Furthermore, the synthesis of multi-lead ECGs needs to take into account the view correlation, while the widespread use of single-lead wearable devices has further highlighted the need to generate a complete 12-lead ECG from a limited number of leads. GANs avoid the need for manual feature engineering through end-to-end learning of the data distribution, thus providing an efficient solution for ECG synthesis. Figure 7 illustrates the three main research directions of ECG synthesis.

In the early stages, many studies focused on generating single-lead and short-duration signals with specific pathological patterns, with the aim of expanding the medical signal dataset. Initially, BiLSTM-CNN GANs proposed by Zhu et al. [49] generated ECG signals on the MIT-BIH dataset, demonstrating excellent performance in terms of the convergence speed of the loss function and the similarity of the shapes. Wang et al. [60] proposed simple-structured fully connected GANs specifically designed to generate heartbeats data of left bundle branch block. Wulan et al. [61] explored three deep learning-based models (WaveNet, SpectroGAN, WaveletGAN) for generating ECG signals containing N, L, and R types of heartbeats, and proposed an SVM-based GAN-train and GAN-test scoring evaluation method.

Subsequently, in the field of multi-lead ECG synthesis, the researchers significantly improved the generation quality through innovative architecture. The 2D BiLSTM GAN model proposed by Zhang and Babaeizadeh [62] successfully synthesized four types of standard 12-lead electrocardiogram signals, including normal, left ventricular hypertrophy, left bundle branch block, and acute myocardial infarction. The success rate of signal synthesis is as high as 98%, and the generated data presents a reasonable physiological state and diverse forms. ME-GAN proposed by Chen et al. [63] can incorporate cardiac disease conditions into specific waveform positions through the Mixup normalization layer, and combine the view discriminator to ensure that 12-lead ECG signals have the correct lead characteristics.

Many studies have also explored the potential application of GANs in lead conversion. Lee et al. [64] used GANs to synthesize chest-lead (V-lead) signals from limb lead (MLII) by R peak alignment technology for the first time, achieving a structural similarity (SSIM) of 0.92. Seo et al. [65] further generated 12-lead ECG from single lead data using the U-net generator, and the Frechet distance (FD) was reduced to 6.701.

Table 2 systematically summarizes the representative research progress of GANs in the field of electrocardiogram signal synthesis in recent years. It covers four aspects: model architecture (such as BiLSTM-CNN, WGAN-GP), generation targets (such as multi-channel synchronization), datasets (such as MIT-BIH, PTB-XL), and evaluation indicators (such as FID, Kappa coefficient, classification accuracy), and presents a review of the key studies in this field over the past six years. The ECG synthesis technology based on GANs has made significant progress. It can generate highly realistic and diverse ECG signals, effectively serving data expansion and algorithm testing. This technology generally adopts the core framework of GANs, namely the adversarial training between the generator G and the discriminator D. However, the specific forms are diverse, including the use of LSTM or BiLSTM to capture temporal dependencies, CNN to extract spatial spectral features, and U-Net to capture detailed features, etc. Training techniques such as gradient penalty and conditional input have also been employed to enhance stability and generation quality. However, the early synthetic signals were unable to generate dynamic heart rate changes and complex pathological rhythms. They also had shortcomings such as insufficient temporal and spatial consistency across different leads and the lack of clinically oriented evaluation indicators. Subsequently, it is necessary to improve the temporal modeling based on the physiological mechanism, establish a multi-channel joint generation framework and a clinical interpretability evaluation system.

#### 4.1.2. Synthesis of EEG

The high cost of EEG data collection and the scarcity of samples limit the application of deep learning models in neuroscience and clinical diagnosis. GANs learn the distribution of real data through adversarial training and generate high-quality synthetic EEG signals, providing an efficient solution for data augmentation.

Hartmann et al. [29] proposed an EEG-GAN framework as early as 2018, which was used to generate EEG signals. This study improved the training strategy of Wasserstein GAN by introducing gradient penalties, thereby enhancing the training stability. The Inception score, Frechet inception distance and sliced Wasserstein distance were used to evaluate this framework. And it was confirmed that the EEG-GAN can generate single-channel samples that are natural and conform to the time–frequency domain characteristics of real EEG. Lee et al. [68] proposed the SIG-GAN framework, which combines the bidirectional long short-term memory network (Bi-LSTM) and the convolutional neural network (CNN), to generate context-aware EEG signal sequences. It effectively fills in the long-term missing data caused by electrode failures in sleep monitoring, while preserving the waveform characteristics of sleep stages. Building on WGAN, Zhang et al. [69] introduced the Conditional Wasserstein GAN (CWGAN) and its multi-generator variant (MG-CWGAN), and began to explore the role of synthetic EEG in classification tasks. However, rather than focusing on classification performance, they placed greater emphasis on the generative aspect.

Table 3 summarizes the applications of GANs in EEG generation. The emergence of GANs has made it possible to apply in the technology of generating brain signals, and it has become a key strategy to solve the problem of scarce EEG data. The research focus has gradually shifted from the generation of single-channel signals to the spatiotemporal relationships of multi-channel signals and the generation of conditions for specific tasks. However, challenges including more refined preservation of the high-frequency features of EEG, enhancement of the cross-subject generalization ability of the generated signals, and verification of the clinical interpretability of the generated data, are all important directions for future research. In line with this trend, recent research in 2025 has moved beyond traditional synthesis to focus on more precise feature extraction. For instance, Avital et al. [70] introduced an automated framework for calculating the average power of EEG signals, significantly enhancing the sensitivity for detecting complex brain activity and behavioral patterns, which provides a more robust foundation for clinical data interpretation.

Among all the generation-based tasks, studies focusing on the quality of ECG have increased significantly over the three-year period from 2020 to 2022. During the subsequent three years from 2023 to 2025, an increasing number of studies began to focus on practical application scenarios, such as analyzing the impact of synthetic data on the improvement of disease diagnosis, including those related to arrhythmia. Unlike the synthesis research of electrocardiogram, most of the related studies on the synthesis of brain waves, electromyography and electrooculogram have clearly mentioned the downstream classification tasks they focus on since their inception. These studies paid less attention to the analysis of signal quality generation and focused more on improving the classification effect. Therefore, in this paper, more literature and its analysis are summarized in the classification section of the later part.

### 4.2. Classification

In the task of analyzing electrophysiological signals, accurate identification and classification are the core goals of physiological state research and disease diagnosis [71]. In classification tasks, the issue of data imbalance is one of the core bottlenecks that restrict the performance of the model. In real scenarios, samples of specific pathological conditions or fine cognitive states are often extremely scarce, while normal signals such as normal heart rhythms and resting-state brain waves are predominant, resulting in classification models being heavily biased towards the majority class and having low sensitivity in identifying the key minority classes [72]. Regarding the problem of severe imbalance in categorical data, although traditional resampling techniques can adjust the sample distribution, they are prone to cause overfitting or information loss, and cannot generate new samples that conform to physiological laws to enrich the feature space [73].

GANs provide an effective data augmentation approach for addressing the issues of data scarcity and class imbalance in the analysis of electrophysiological signals [74]. The generator simulates the real data distribution to provide diverse training samples for the classifier, effectively alleviating overfitting. The discriminator discovers deep discriminative features and simultaneously optimizes the clarity of the classification boundary and the robustness against noise during adversarial training [75,76]. In recent years, researchers have developed various GANs-based frameworks that use the generation of realistic electrophysiological signals to expand the training data and apply it to downstream classification tasks [77]. Although these studies differ in their frameworks, scenarios and evaluation methods, their common goal is to leverage the generative capabilities of GANs to break through data limitations and significantly improve classification performance [78].

#### 4.2.1. Classification of ECG

After exploring the generation effect of ECG, more innovative architectures of GANs emerged from 2023 to 2025, achieving significant breakthroughs in the application field of electrocardiogram signal classification. By optimizing the data distribution and dynamic generation mechanism, the ECG-GAN technology has significantly enhanced the practicality of machine learning in the field of ECG medical diagnosis.

In the exploration of model architecture, Rafi and Woong Ko [79] combined multi-head attention with adversarial generation and achieved a classification accuracy of 99.67% on MIT-BIH, which was 7.2% higher than that of traditional CNN. Xia et al. [80] proposed TCGAN, which combines the generator of the Transformer to generate samples of the minority class of heartbeats. This enabled the CNN to achieve an accuracy rate of 94.69% in classifying the four types of heartbeats on the MIT-BIH dataset. Based on this, Zhou and Huang [81] combined the Transformer and LSTM branches to propose a dual-branch GAN (DB-GAN), which generated 12-lead ECG signals that retained pathological features. This resulted in an increase from 90.98% to 96.66% in the classification accuracy of the four types of diseases on the MIT-BIH database, and the recall rate for ventricular premature beats was improved by 26%. Kuntalp and Duzyel [32] innovatively incorporated t-SNE to analyze the data cluster structure and independently trained generators for multiple cluster categories, thereby increasing the F1 score of the KNN classifier for the combined heartbeat data by 15%.

In terms of the generation mode, building on the research of converting single-lead ECG to multi-lead ECG, Yoon and Joo [82] proposed a method using pix2pix GAN to generate 12-lead signals from Lead I. This resulted in an accuracy rate of 96.33% for cardiovascular disease classification on the PTB-XL database, surpassing the 94.12% achieved by real multi-lead data.

Table 4 systematically summarizes the representative research progress of GANs in the field of electrocardiogram signal classification in recent years. It conducts a comparative analysis of key studies in the field from four dimensions: model architecture, generation objective, dataset, and evaluation indicators. In summary, a hybrid architecture and conditional generation framework are commonly adopted in the research of application of GANs in the field of ECG classification. Combining them with LSTM, attention mechanisms, and Transformers can enhance the fidelity and diversity of the synthesized data, thereby addressing the issues of data scarcity and imbalance [4,83,84,85,86,87,88]. The experimental dataset is mainly based on MIT-BIH. The evaluation metrics mainly include classification accuracy, recall rate, F1 score, etc., which confirm the practicality of data augmentation. However, the fidelity of the generated signals is often limited by the simplification of the model architecture, while complex models have high computational costs. Most studies rely on a single dataset and lack cross-database validation, resulting in insufficient generalization ability of the models. Furthermore, the generated signals may introduce noise or deviations, thereby affecting the reliability of downstream tasks. Future research should focus on developing lightweight GANs models to reduce computational costs, strengthening clinical validation, evaluating the consistency of the application of generated signals through large-scale multi-center trials, and exploring unsupervised learning to reduce reliance on labeled data.

#### 4.2.2. Classification of EEG

Electroencephalogram (EEG) signals, due to their high temporal resolution and non-invasive feature, are widely used in various classification tasks such as emotion recognition, fatigue detection, and diagnosis of neurological disorders. However, EEG data often encounter challenges such as scarce samples, imbalanced categories, and individual differences, which limit the generalization ability of deep learning models. GANs enhance the training set by generating synthetic data, thereby becoming a key technology for solving the aforementioned problems.

In the field of emotion recognition, Tian et al. [89] and Qiao et al. [90] respectively developed the dual-encoder VAE-GAN and an innovative self-supervised data augmentation strategy, named SSDAS-EER. Through spatio-temporal feature modeling and generation of cross-subject event-related potentials, they separately achieved an accuracy rate of 97.21% and 97.27%.

In fatigue detection, the GDANN model proposed by Zeng et al. [91] and the GAN-GCN architecture proposed by Ardabili et al. [92] were combined to enhance the accuracy of cross-subject fatigue classification and maintain robustness in noisy environments. In sleep staging direction, Zhou et al. [93] and Kuo et al. [94] respectively utilized GAN combined with Gaussian white noise and self-attention mechanism to effectively alleviate the imbalance problem of sleep stage data and improve the accuracy of personalized sleep scoring.

The research over the past three years has focused more on technological integration and framework innovation. Gu et al. [95] were the first to propose Domain Generation Graph Network (DGGN) in 2023. By integrating graph convolution (GCN) with LSTM to dynamically model the spatio-temporal topological relationships of electroencephalogram channels, it laid the foundational architecture for cross-subject generalization. Gilakjani et al. [96] introduced a contrastive learning mechanism to enable the new architecture to utilize graph networks to align the feature distributions of different subjects, thereby breaking through the generalization bottleneck caused by individual differences. Qiao et al. [90] designed the self-supervised strategy (SSDAS-EER), which guides the GANs to learn key spatial-spectral features through masked spectral fusion, addressing the sparse representation problem of the original EEG. A variety of application scenarios and innovative architectures have jointly driven the development of electroencephalogram analysis towards higher accuracy and stronger generalization capabilities.

Table 5 presents the research that explores the application of GANs in EEG generation. Overall, the generated EEG images are applied in fields such as visual perception, sleep stage classification, emotion classification, and fatigue driving detection. In various application scenarios, conditional generation (such as CWGAN) and Wasserstein optimization (WGAN-GP) have become the mainstream approaches. The architecture design has evolved from the basic DCGAN to a composite model that combines CNN, RNN and GNN. The evaluation of the model usually takes into account various aspects such as generation quality (like FID) and improvement in classification tasks (such as classification accuracy). However, although there have been numerous studies focusing on the enhancement and classification of EEG signals, most of the existing methods are limited to specific tasks and lack in-depth exploration of the generalization issues across modalities and populations. The fidelity of generating multi-channel signals over a long time period, the modeling capability for complex spatiotemporal characteristics and the lack of assessment criteria for time series data remain significant challenges that need to be overcome.

#### 4.2.3. Classification of EMG and EOG

GANs have demonstrated potential in multiple applications of biological signal processing such as EMG and EOG, and have gradually become effective tools for data augmentation and simulation to address the scarcity of such signal data and related challenges in multiple application scenarios.

Chen et al. [52], Zhang et al. [100], and Mendez et al. [101] utilized DCGAN/EBGAN to generate multi-channel EMG data, thereby improving the accuracy of gesture classification. The research conducted by Chen et al. demonstrated that synthetic data shares similar statistical characteristics with real data, and histogram equalization was employed to optimize the classification performance. However, Mendez et al. pointed out that the characteristic distributions of the generated signals by GANs, such as MAV and RMS, showed significant differences from the real data when tested by the Mann–Whitney U test (*p* < 0.05), and post-processing re-scaling was necessary to enhance their usability. Zanini and Colombini [102] utilized DCGAN combined with style transfer to simulate the tremor patterns of Parkinson’s patients and successfully extended it to different movement protocols, highlighting the flexibility of GANs in medical data simulation. Jiao et al. [103] utilized CWGAN to enhance EOG data, integrated EEG data, and combined with LSTM to detect the driver’s fatigue state. The classification accuracy reached 98%, demonstrating the potential of GANs in multi-signal fusion.

GANs have expanded from basic data augmentation to scenarios such as security attack and defense, disease simulation, and real-time monitoring in the fields of EMG and EOG. Their core value lies in addressing the bottleneck of obtaining biological signals. Table 6 summarizes and reviews these studies over the past few years. All the studies have verified the effectiveness of synthetic data. EBGAN and CWGAN have improved upon traditional GAN and WGAN by incorporating the concept of energy or imposing conditional constraints, demonstrating superior performance in feature space learning and pattern stability. Fast Neural Style Transfer significantly speeds up the style transfer process, making it possible for real-time applications. However, the physiological rationality of the generated data, the ability to generalize across different scenarios, and the standardization of evaluation remain key challenges. The stability of GANs training and the problem of mode collapse still need to be overcome. In the future, it is necessary to explore cross-subject generative frameworks, physiological constraints in GANs loss functions, and standardized evaluation protocols to promote the reliable application of synthetic biological signals in clinical and industrial settings.

**Figure 8:**
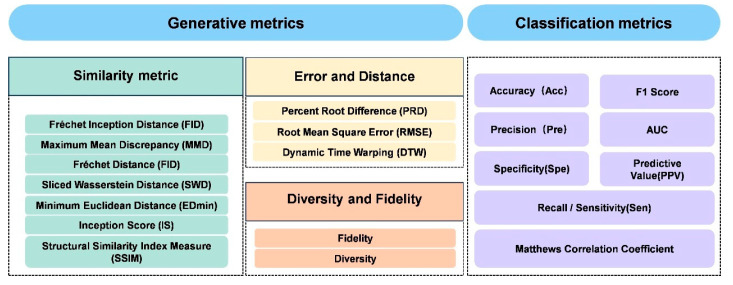
Commonly adopted quantitative metrics for GAN-based generative and classification tasks.

The recent development trend of GANs in the application research of electrophysiological signals is to combine GANs with transfer learning, graph neural networks and other network models. Then, domain adversarial training is used to reduce the distribution differences, and the generated data is utilized to indirectly improve the performance of the classifier. This type of research has shifted from single data augmentation to an end-to-end holistic architecture, with a focus on practical application verification in real scenarios. The generated data has solved the data imbalance problem of the classification model. The improvement in the model’s classification performance, in turn, validates the quality of the generated data and the effectiveness of data augmentation. Therefore, the synthesis and classification of data are essentially inseparable tasks. We summarize the evaluation metrics for such tasks in Figure 8. Crucially, a common methodological pitfall in this domain is “data leakage” during subject-specific splitting; if segments from the same subject are split across training and test sets, the classification accuracy—used here as a proxy for generation quality—will be artificially inflated. Furthermore, researchers should avoid “metric–task mismatch” by ensuring that generative fidelity is not evaluated solely through statistical similarity, but also through the preservation of diagnostic features. Despite these advancements, the adversarial mechanism of GANs may still introduce training instability, and the contribution of the generated data to classification performance lacks an explainable analysis. In the future, it is necessary to explore more stable training frameworks for adversarial attacks, and combine studies on physiological feature interpretability to enhance the credibility of the model.

### 4.3. Denoising

During the acquisition process, electrophysiological signals are frequently contaminated by various noise sources [104]. These sources include Power Line Interference (PLI), Baseline Wander (BW), Electrode Motion (EM), Muscle Artifacts (MA), and Random Noise (RN). These noise sources significantly degrade signal quality, which in turn undermines the accuracy of subsequent data analysis and diagnosis [105]. The denoising process enables the extraction of valid information from noise-contaminated signals, facilitating more accurate analysis and interpretation of electrophysiological recordings. Therefore, denoising electrophysiological signals remains an important and challenging problem [106].

GANs offer significant advantages for denoising electrophysiological signals [107]. By learning the mapping between noisy and clean signals, GANs can effectively simulate realistic noise, thereby enhancing their ability to capture the characteristics of actual interference encountered in practical applications [108]. Furthermore, GANs are capable of generating high-quality denoised samples by extracting these feature mappings while preserving the inherent information within the signals, ultimately resulting in improved signal fidelity. In recent years, GANs have demonstrated significant potential in biomedical signal denoising, particularly for electrocardiogram (ECG) and electroencephalogram (EEG) data [109]. Table 7 systematically summarizes the key techniques and performance metrics of representative recent studies, providing quantitative benchmarks for future research.

Early efforts to incorporate adversarial learning into ECG denoising were initiated by Wang et al. [110], who introduced a framework that optimized noise distribution modeling through a loss function integrating both global and local feature representations. Building upon this, Xu et al. [111] developed adversarial residual networks with skip connections to further improve denoising robustness. More recently, Wang et al. [112] proposed a hybrid architecture combining conditional generative adversarial networks (cGANs) with convolutional autoencoders, achieving an average signal-to-noise ratio (SNR) exceeding 39 dB, while preserving clinically significant waveform characteristics.

Concurrently, EEG denoising research has trended toward multi-technology integration. Yin et al. [109] proposed GCTNet, an innovative framework that integrates CNNs with Transformer architectures, leveraging a GAN-guided parallel network to capture complex spatiotemporal dependencies and reconstruct clean EEG signals. Similarly, Cai et al. [113] introduced DHCT-GAN, a dual-branch hybrid model whose superiority in artifact removal was quantitatively validated. Addressing noise of a specific origin, Lin [114] designed BCGGAN to mitigate ballistocardiogram (BCG) artifacts in EEG-fMRI recordings. In addition, Sikkandar [115] combined brain storm optimization with VAE-GAN to achieve precise suppression of EEG artifacts, reaching an accuracy of 98.5%.

The expansion of application scenarios further underscores the value of GANs in denoising electrophysiological signals. Sawangjai et al. [55] introduced EEGANet, which removes oculomotor artifacts without requiring additional electrooculogram (EOG) channels, while Brophy [116] addressed the need for real-time denoising in brain–computer interfaces. Tibermacine et al. [117] compared standard GANs with Wasserstein GANs with gradient penalty (WGAN-GP), thereby identifying the contexts in which each adversarial training strategy is most effective.

Notably, multimodal fusion has emerged as a promising direction. De et al. [118] developed the GLEAM framework to fuse EEG and surface electromyography (sEMG) signals for pain detection. Mai et al. [119] combined facial expression analysis with denoised behind-the-ear EEG to construct a lightweight fatigue warning system.

**Figure 9:**
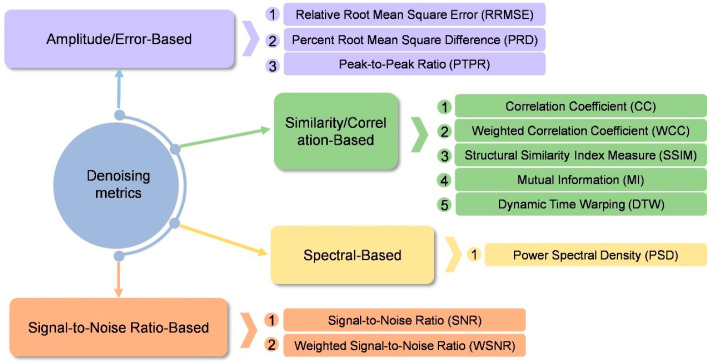
Commonly adopted quantitative metrics for GAN-based denoising tasks.

Collectively, the studies summarized in Table 7 demonstrate that recent architectural innovations-such as dual-branch designs and residual connections-have been instrumental in enhancing model generalization. Improvements in adversarial training strategies, including the incorporation of multiple discriminators and modifications to adversarial feature-loss functions, have addressed the critical challenge of preserving signal detail during denoising. Commonly adopted quantitative metrics for GAN-based denoising tasks are summarized in Figure 9. Performance indicators such as SNR, relative root-mean-square error (RRMSE), and classification accuracy consistently demonstrate that GAN-based models significantly outperform traditional methods, including wavelet thresholding and independent component analysis (ICA), thereby offering a reliable solution for real-time clinical applications. While metrics like SNR and RRMSE provide objective measures, a significant pitfall is the over-reliance on visual inspection of the denoised waveform. Visual clarity does not always guarantee the removal of subtle, high-frequency artifacts that could distort subsequent clinical analysis.

Future research should focus on two key directions. First, lightweight model deployment is essential, with promising approaches such as An’s [120] sample entropy-based threshold normalization method showing potential for reducing computational complexity. Second, improving cross-modal generalizability remains a major challenge. Inspired by the design of BrOpt_VAGAN proposed by Sikkandar et al. [115], which combines brainstorm optimization with a variational autoencoder GAN, future studies may explore similar hybrid frameworks to achieve robust artifact suppression across heterogeneous EEG applications.

**Table 7:** Electrophysiological denoising with GANs.

Study	Modality	Dataset	Architecture	Result Evaluation
[109]	EEG	MIT-BIH; Semi-simulated EEG/EOG dataset; Real Data	GCTNet GAN Architecture, Generator: Transformer + CNN	RRMSE: 0.340 + 0.044; CC: 0.929 ± 0.015 SNR: 11.123 ± 1.306; WSNRe: 16.705 ± 1.251 WSNRh: 22.104 ± 1.297; WCCe: 0.911 ± 0.016 WCCr: 1.074 ± 0.041
[2]	ECG	MIT-BIH	LSGAN	SNR: 16.30, MSE: 0.0016, PRD: 9.63
[110]	ECG	MIT-BIH	GAN (loss function Improve)	SNR: 33.02, RMSE: 0.944
[117]	EEG	self-collection	GAN, WGAN-GP	SNR: 13.03, PRMSE: 0.908, MI: 5.01, 5.07 SD: 3.36, MAE: 0.092, 0.108, PSNR: 18.65 CC: 0.86, DTW: 10.43
[111]	ECG	self-collection	GAN, ResNet (discriminator)	SNR: 60.5719, RMSE: 0.0073
[112]	ECG	MIT-BIH	CGAN (add CAE in Generator)	average SNR: 44 dB
[120]	EEG	HaLT	GAN (With SETET Method)	RMSE: 0.0757
[114]	EEG-fMRI	self-collection	BCGGAN (Base on CycleGAN)	PTPR: 1.203
[116]	EEG	PhysioNet EEGdenoiseNet	GAN	Co-sine similarity: 0.998 on EMG-score
[113]	EEG	EEGdenoiseNet MIT-BIH semi-simulated EEG	DHCT-GAN (Hybrid CNN–Transformer)	RRMSEt: 0.3975, RRMSEf: 0.2904 CC: 0.9184, SSIM:0.6996, MI: 1.0159
[55]	EEG	self-collection	EEGANet	APCC: 0.141 ± 0.057. RMSE: 1.835 ± 0.550
[119]	Ear-EEG	self-collection	GAN	RRMSEt: 0.210, RRMSEf: 0.161
[115]	EEG	CHB-MIT, KAU	Variational Autoencoder GAN	12.3–12.98% On EEG + muscle signal artifact

### 4.4. Anomaly Detection

GANs can be used for anomaly detection of electrophysiological signals. Abnormality detection is one of the important tasks in the field of electrophysiological signal processing, aiming at identifying signals that are significantly different from the normal pattern and may indicate potential diseases, abnormalities or other important information. While traditional methods often rely on large amounts of labeled data or preset thresholds, GANs can provide innovative solutions through unsupervised learning mechanisms.

The core concept of using GANs for abnormal detection in electrophysiological signals involves training a generator to model the distribution and characteristics of normal electrophysiological signals, while a discriminator evaluates new and unseen signals identify anomalies by determining whether they deviate from the learned distribution. This approach enables effective anomaly detection even in the absence of clearly labeled abnormal samples.

The following subsection examines GAN-based anomaly detection approaches for electrophysiological signals, organized according to the targeted disease types.

#### 4.4.1. Epilepsy Detection

Epilepsy, as an acute neurological disorder, requires the detection of transient abnormal discharges and preictal states in EEG signals. Detecting these events is crucial for timely clinical intervention. Traditional approaches are limited by the dynamic brain network modeling capabilities and the scarcity of preictal samples, while GANs effectively address these challenges by generating synthetic data with unsupervised learning.

In the field of continuous epilepsy monitoring, You et al. [121] pioneered the development of an unsupervised behind-the-ear EEG detection framework, incorporating Gram matrix enhancement to improve anomaly localization, achieving a sensitivity of 96.3% and a false positive rate of 0.14 per hour. However, this method has limitations in capturing time-varying functional connectivity. For preictal prediction, Xu et al. [122] innovatively utilized GANs to generate multi-channel preictal EEG signals, achieving a 5% improvement in prediction accuracy through data augmentation. A recent breakthrough came from Abdi-Sargezeh et al. [123], who designed a VAE-cGAN cross-modal mapping model to convert scalp-mounted EEG (scEEG) to intracranial EEG (iEEG), improving the accuracy of interictal epileptiform discharges (IEDs) detection to 76%, a 3–11% increase over traditional models. Additionally, the semi-supervised feature learning, unsupervised data augmentation, and cross-modal fusion strategies proposed by Truong et al. [124], Usman et al. [125], and Gao et al. [126] have effectively addressed key challenges faced by GANs in epilepsy monitoring, such as data scarcity and modality differences, providing valuable insights for future research.

#### 4.4.2. Arrhythmia Detection

Arrhythmia is an abnormal disturbance of the heart’s electrical activity. It can result in abnormal heartbeat rhythms and, in severe cases, may lead to heart failure, stroke, or other complications. As the gold standard for recording cardiac electrophysiological activity, ECG provides a visual representation of arrhythmia types and severity through various waveform characteristics, such as P waves, QRS complexes, and ST segments. However, traditional ECG analysis heavily relies on the expertise of clinicians. Additionally, the limited availability of abnormal rhythm samples and the issue of class imbalance hinder the generalization of supervised learning models. GANs have gradually addressed these challenges by generating realistic ECG samples and refining feature representations.

In early studies, to optimize the unsupervised detection performance, Shin et al. [127] improved the loss balancing mechanism of AnoGAN [128] to detect ECG anomalies by decision boundary optimization with an AUROC of 0.9475, which solved the problem of subjective threshold setting. Building on this, Wang et al. [5] proposed a two-stage hierarchical framework MadeGAN, which integrates memory-enhanced self-encoder and migration learning. The first level reconstructs normal ECG patterns through memory modules, and the second level uses discriminator features for arrhythmia classification. This framework achieved a recall rate of 96.4% on the MIT-BIH dataset. More recently, Xing et al. [129] proposed an improved VAE-GAN framework based on time-series prediction, where the variational decoder predicts future sequences instead of reconstructing the inputs, demonstrating state-of-the-art performance on the ECG5000 and MIT-BIH ECG datasets as well as railway track scan images. The breakthrough in temporal modeling came from Qin et al. [130] with ECG-ADGAN, whose core innovation lies in embedding a Bi-LSTM layer within the generator to capture long-range dependencies. This model achieved an AUC of 95.9% for detecting unknown arrhythmias, highlighting the importance of temporal constraints in ECG synthesis.

#### 4.4.3. Depression Detection

Depressive Disorder is the most common mental health disorder worldwide, and recent studies have explored the use of EEG biomarkers processed with machine learning algorithms for symptom detection, yielding promising results. However, the generalizability of these models is limited by the small datasets of each category. Carrle et al. [131] employed a conditional WGAN architecture to generate EEG time-series data from patients with depression and healthy controls. By optimizing data distribution alignment through a CNN-based generator and discriminator, their approach enhanced sample augmentation for depression detection.

Adolescent depression with non-suicidal self-injury (NSSI) exhibits only subtle differences in EEG features and suffers from a scarcity of clinical labels. To tackle these challenges, Liang et al. [132] proposed NSSI-Net, a multi-conceptual GAN framework. The model jointly optimizes a spatiotemporal feature-extraction module and a four-branch discriminator—each branch focusing on signal, gender, domain, or disease. Compared with the baseline, NSSI-Net improves abnormality-detection accuracy by 5.44%, effectively addressing the problem of extracting generalizable high-dimensional EEG features.

#### 4.4.4. Sleep Apnea Detection

Sleep Apnea Syndrome (SAS) manifests as respiratory event–related rhythmic changes in ECG signals; however, manual annotation of these events is both costly and time-consuming.

To address this challenge, Shen et al. [133] proposed 1D-ConReNet, a self-supervised multi-task framework. This model achieves 89.25% segment-detection accuracy on the Apnea-ECG dataset. A key innovation of 1D-ConReNet is the transfer of generative weights into both its GAN and CNN modules, which significantly enhances generalization performance under small-sample conditions.

Table 8 provides a comprehensive summary of recent studies in the field of anomaly detection. It is evident that the methods listed rely on the strong performance of GANs in the field. Categorized by signal modality, in the ECG domain, the focus is primarily on arrhythmia and respiratory monitoring, while the EEG domain is primarily concerned with epilepsy and psychiatric disorders. From the perspective of GAN architectural innovations, unsupervised learning has demonstrated substantial advantages in these applications, with particular attention given to time-series prediction and long-range dependency modeling.

Regarding the evaluation of such models, since anomaly detection is frequently framed as a binary classification task, the adopted metrics largely overlap with the classification tasks discussed in Section 4.2. As summarized in Table 8, standardized evaluation should prioritize metrics such as Accuracy, F1-score, and AUC-ROC, while providing a detailed breakdown of Sensitivity (Recall) and Specificity to capture the model’s diagnostic performance. However, a frequent methodological pitfall in this task is the imbalance of evaluation in highly skewed medical datasets, where a high overall accuracy may mask a model’s failure to detect rare but critical clinical events. Therefore, the use of G-mean and Precision–Recall analysis (e.g., AUPRC) is highly recommended to ensure reliable detection and minimize clinical false alarms. By adopting these rigorous assessment standards, GANs have significantly reduced the models’ reliance on anomalous data, leading to notable improvements in both sensitivity and specificity for anomaly detection, particularly in the handling of high-dimensional, time-varying EEG signals.

### 4.5. Modal Transformation and Fusion

GANs have become an ideal tool for dealing with multimodal electrophysiological signal conversion and fusion by virtue of their powerful generative capabilities and the advantages of modeling complex data distributions. Their core advantage lies in the ability to synthesize virtual data that capture intricate associations across different modalities, thereby enabling effective cross-modal mapping and alleviating the challenge of acquiring real data in certain target modalities. For instance, GANs are capable of synthesizing PPG signals based on ECG data. In specific applications such as visual stimulus reconstruction, GANs are employed to convert low-resolution or abstract electrophysiological signals into more interpretable image representations, thereby deepening the understanding of underlying neural processes. These applications bring new possibilities in the fields of neuroscience research and clinical diagnosis.

In 2021, Cheng et al. [136] proposed the BMT-GAN, a cross-modal framework that converts EEG into fMRI images to assist medical diagnosis. This work initially demonstrated the potential of GAN to enhance the analytical capability of one modal by leveraging data from another. In the same year, Shin et al. [137] employed a GAN to synthesize photoplethysmogram (PPG) signals from ECG, addressing the scarcity of PPG data and introducing a novel strategy of data augmentation for unimodal PPG analysis. To further expand the application of cross-modal conversion, Dissanayake et al. [138] proposed a Pix2Pix-GAN architecture based on U-Net to transform phonocardiogram (PCG) signals into clinically meaningful ECG features, significantly enhancing the convenience of cardiac function monitoring. In a recent study, Li et al. [139] introduced the first GAN-based framework for Mandarin speech reconstruction, which directly generates speech waveforms from facial and cervical sEMG signals, marking a breakthrough in assisted communication for laryngectomized patients.

Despite these promising developments, the generalizability of these cross-modal transformations, their robustness across diverse pathological states, and the clinical validity of the synthesized signals all warrant further investigation.

In the field of modality fusion, Shen et al. [56] introduced the CrossGAN. This framework simultaneously integrates EEG and image modalities to project brain responses into the stereoscopic image quality ranking (SIQR) task, thereby enabling cross-modal joint representation learning.

In recent years, a particularly challenging research direction has been the reconstruction of visual stimulus images from EEG signals. Khaleghi et al. [140] proposed a geometric deep network-based GAN (GDN-GAN) to generate visual saliency maps and grayscale images by mapping EEG features into the image domain via a graph convolutional network. Similarly, Deng et al. [141] and Mishra et al. [142] used an IC-GAN [143] and NeuroGAN to achieve EEG-to-image reconstruction, respectively. These studies demonstrate the potential of inferring visual content from EEG. A shared challenge across these works lies in handling the inherently noisy and information-sparse nature of EEG signals, as well as in accurately modeling the high-dimensional mappings between neural activity and complex visual representations.

The studies discussed in this section, together with related work, are summarized in Table 9. Overall, GANs have emerged as a powerful framework for multimodal processing of electrophysiological signals, owing to their capacity for data augmentation, modality conversion, and cross-modal representation learning. Cross-physiological signal conversion approaches exploit the inherent correlations among diverse biosignals to enable integrated analysis, while visual reconstruction efforts aim to bridge the gap between signal domains and the visual domain.

Standardized evaluation for these cross-modal tasks requires a multi-dimensional approach. As evidenced by the metrics in Table 9, fidelity is often assessed using MSE, PRD, and Pearson Correlation Coefficient (CC), while structural and perceptual similarity in visual reconstruction tasks are measured via SSIM and Peak Signal-to-Noise Ratio (PSNR). For conversion tasks involving semantic categories, classification-based metrics like Kappa coefficients and F1-scores are essential to ensure the preservation of diagnostic information. A common pitfall in modality translation is the “semantic drift,” where the generated signal appears visually or statistically plausible but loses its original physiological meaning. Over-reliance on qualitative visual results can be misleading; therefore, quantitative consistency checks, such as Mel-Cepstral Distortion (MCD) for speech-related biosignals, should be prioritized to validate the objective accuracy of the transformation. Nevertheless, challenges such as the authenticity of translated data, the quality of reconstructed outputs, and the interpretability of generative models remain major bottlenecks in the application of GANs for modality translation in electrophysiological signal analysis.

### 4.6. Other Applications

In recent years, GANs have gained increasing attention in the field of bio-electrical signal processing, demonstrating potential in several specialized areas beyond the primary applications discussed earlier. However, due to the relatively limited number of studies in these areas, this section categorizes and summarizes them into four key themes: BCI applications, signal de-identification, feature extraction, and signal reconstruction.

#### 4.6.1. BCI Applications

In BCI research, EEG data acquisition is often hindered by inter-subject variability and high experimental costs, resulting in limited training datasets and poor model generalization. GANs have proven effective in addressing data scarcity by generating high-fidelity synthetic EEG signals.

For example, Zhang et al. [144] employed a cGAN to convert theoretically simulated EEG into empirical training data, yielding a 2.17% improvement in classifier accuracy. Similarly, Li et al. [145] and Xu et al. [146] each developed GAN-based frameworks for motor imagery EEG synthesis, targeting healthy subjects and stroke patients, respectively. More recently, Sarikaya et al. [147] applied heterogeneous adversarial transfer learning (HATL) to emotion-recognition scenarios, reducing required calibration time by 30%.

These methods are summarized in Table 10. All the studies demonstrated promising performance in downstream tasks. A central challenge, however, lies in ensuring that the generated signals preserve critical physiological features such as event-related desynchronization (ERD) and event-related synchronization (ERS), while avoiding overfitting due to distributional discrepancies between synthetic and real data.

#### 4.6.2. De-Identification

Clinical electrophysiological signals often contain sensitive personal information and thus be subject to strict privacy regulations. Traditional anonymization techniques tend to degrade the diagnostic utility of these recordings. In contrast, GANs can generate synthetic data that retain similar statistical characteristics without revealing personal identity, thereby achieving a better balance between privacy protection and data utility.

Piacentino et al. [36] were the first to propose converting dynamic ECG sequences into time–frequency images as inputs to a GAN, enabling the generation of synthetic ECGs that preserve temporal characteristics for anonymization purposes. Building on this, Jafarlou et al. [149] combined cGANs with an identity loss function to filter out personally identifiable information while preserving the diagnostic utility for arrhythmia detection. Kang et al. [150] were the first to reveal the threat posed by synthetic EMG signals to biometric identification systems, emphasizing that biometric systems need to consider defense mechanisms against synthetic data. Further advancing this line of work, Kang et al. [151] introduced a multi-task learning framework to simultaneously perform arrhythmia classification and identity obfuscation. In parallel, Pascual et al. [152] and Thambawita et al. [153] applied privacy-preserving GAN methods to epileptic EEG activity and ECG data, respectively. In the latest research, Zarean et al. [154] developed a synthetic EEG biometric authentication system resilient to adversarial attacks that remains robust against adversarial use of GAN-generated signals.

The aforementioned studies are collectively summarized in Table 11. Overall, GAN-generated data can eliminate personally identifiable information while maintaining similar statistics and feature distributions to real data, which provides a safer and more compliant alternative for research and application.

#### 4.6.3. Feature Extraction

Bio-electric signals are often contaminated by physiological information from multiple sources (e.g., fetal ECG embedded in maternal ECG), and traditional filtering methods struggle to isolate the target features with high accuracy. GANs can achieve end-to-end target feature extraction by virtue of their powerful feature-decoupling capabilities. Based on this, GANs are employed to separate target features from complex electrophysiological signals.

Yao et al. [155] proposed a CycleGAN-based autoencoder structure that filtered alcohol-related information after converting the EEG signal into images, incurring only a 6.2% loss in stimulus information accuracy. Zhong et al. [54], on the other hand, combined the short-time Fourier transform (STFT) with a GAN to extract fetal electrocardiographic (FECG) signals from maternal abdominal-lead ECGs directly in the time–frequency domain, achieving high-sensitivity separation. Similarly, Xiao [156] extracted respiratory signals from non-contact capacitive coupling electrocardiograms (cECG), using a Time–Frequency Domain GAN (TF-GAN), and 86.3% of the generated signals were highly correlated with reference signals, verifying the feasibility of cross-modal feature extraction.

Related studies in the feature extraction category are summarized in Table 12. As shown in Table 12, these methods significantly improve the accuracy of cross-modal feature separation while preserving key physiological features, providing a new paradigm for complex bio-electric signal parsing.

#### 4.6.4. Signal Reconstruction

Electrophysiological signals are often affected by equipment limitations during acquisition. They may have low sampling rates and be corrupted by environmental noise, leading to poor signal quality. Traditional reconstruction methods struggle to recover high-fidelity waveforms. In recent years, studies have shown that GANs, with their strong generative ability, can reconstruct high-quality signals from low-quality inputs while preserving key physiological features.

Luo et al. [157] developed a WGAN incorporating a Time–Space–Frequency (TSF-MSE) loss function to reconstruct high-fidelity signals from low-sampling-rate EEG data. The reconstructed signals demonstrated improved classification accuracy on the AO, GAL, and MI datasets. Chen et al. [158] employed an Attention-based Spectral Normalization GAN (Att-SNGAN) to reconstruct high-fidelity ECG signals from BCG data, preserving the temporal dynamics required for heart rate variability (HRV) analysis and obviating the need for beat-cycle pre-segmentation.

This class of research is summarized in Table 13. In summary, GANs are able to learn the intrinsic distributional properties of signals through adversarial training of optimization generators, providing quantifiable performance improvements for biosignal reconstruction. Moreover, to address the computational bottlenecks often associated with generative architectures, newer algorithmic directions have emerged in 2025. A notable example is the work by Avital et al. [159], which proposed a CNN-based alternative to the conventional Independent Component Analysis (ICA). This method achieves high-fidelity brain activity analysis with significantly reduced latency compared to traditional iterative tools like EEGLAB, representing a pivotal shift towards fast and scalable real-time signal reconstruction pipelines.

This section synthesizes four categories of emerging applications of GANs in electrophysiological signal processing. Overall, the GAN research presented in this section demonstrates multifaceted value in bio-electrical signal processing: at the data level, addressing data scarcity and privacy preservation; at the feature level, achieving high-precision separation of signal components; and at the quality level, enabling signal reconstruction to enhance usability. The core challenge in these applications lies in balancing the authenticity of generated data, privacy preservation, and downstream task performance. In the future, cross-modal generation and lightweight model deployment may be further explored.

## 5. Discussion

GANs, as powerful deep learning models, have demonstrated significant potential and unique advantages in the field of electrophysiological signal processing. By reviewing the existing research, we found that GANs not only have successfully been applied to data synthesis, effectively alleviating the common problems of data scarcity and class imbalance in medical research, but also have achieved remarkable results in multiple aspects such as signal denoising, feature extraction, anomaly detection, and cross-modal transformation. In the following sections, we further discuss the critical challenges for clinical translation and the emerging potential applications of these models.

### 5.1. Clinical Realism and Practical Challenges

GANs have demonstrated significant potential in electrophysiological signal processing, but their clinical translation faces critical hurdles regarding physiological plausibility and generalization. Unlike natural images, synthetic electrophysiological signals must adhere to strict biological constraints; for instance, synthetic ECG must preserve the precise temporal-spectral relationships (e.g., P-QRS-T complex) to be diagnostically valid [14,156]. Current validation often relies on expert visual inspection or downstream classification accuracy, yet there is a growing need to enforce biological priors directly within the loss functions to ensure high-fidelity reconstruction [157].

Furthermore, the generalization of GAN models remains a bottleneck. Strong performance on a single centralized dataset often fails to translate to real-world clinical settings due to cross-subject, cross-device, and cross-dataset variability [3,29]. Inherent training instabilities, such as mode collapse, further limit the diversity of generated samples, potentially leading to overfitting on specific noise profiles rather than learning robust physiological patterns. Integrating Explainable AI (XAI) and enhancing model transparency are therefore crucial steps toward establishing clinical trust and ensuring that GAN-based tools can generalize across diverse populations and recording environments.

Establishing such trust requires a granular understanding of how GAN performance varies across different physiological domains. To provide a clearer perspective on the current landscape, Table 14 presents a comparative summary of GAN applications across the four primary electrophysiological modalities, marking their respective maturity levels, data availability, typical tasks, and leading model architectures. This comparison highlights the significant gap between established signals like ECG and EEG versus emerging or nascent modalities such as EMG and EOG, underscoring the specific methodological hurdles and open gaps that remain for each modality in real-world medical environments.

### 5.2. Future Frontiers and Strategic Directions

Apart from the relatively mature applications, the potential of GANs in the field of electrophysiological signals is being further explored. Potential applications can be explored in the future include but are not limited to: (1) Signal prediction: G can learn historical patterns to predict future signal values, which has important implications for predicting disease progression; (2) Signal segmentation: GANs can automate and refine the segmentation of brain waveforms or cardiac rhythms, aiding medical diagnosis; and (3) Personalized medical applications: solutions can be customized based on patient characteristics to provide auxiliary treatment. These potential applications will further enrich the research content of GANs in electrophysiological signal processing and provide more innovative solutions for the development and clinical practice of electrophysiology.

Although GANs have broad application prospects, their applications in the processing of electrophysiological signals still face many challenges. Firstly, the inherent training instability of the model, such as mode collapse, limits the diversity of the generated signals. Secondly, the limited data volume in the medical field can easily lead to overfitting and poor generalization ability. Thirdly, there is currently no comprehensive evaluation framework that adequately reflects practical utility, making it difficult to compare and assess the quality of generated signals. Finally, the black-box nature of the model leads to poor interpretability, coupled with the high consumption of computing resources, together forming the main obstacles to its promotion in terms of security and practicality.

To overcome these challenges and fully unlock the potential of GANs, future research should concentrate on the following directions. Firstly, the improvement and innovation of GANs models to solve problems such as its stability, convergence, and resource consumption need further study. Secondly, more complex tasks such as multimodal data fusion and cross-domain signal conversion to promote the deepening and expansion of GANs in electrophysiology need to be explored. Exploring the combination of GANs with other technologies (such as semi-supervised learning, transfer learning, and reinforcement learning) holds the promise of further enhancing their performance in the analysis of electrophysiological signals. In addition, interdisciplinary cooperation will be the trend, combining professional knowledge in electrophysiology with deep learning algorithms to jointly promote the development of personalized medicine. Meanwhile, integrating explainable AI (XAI) techniques to enhance model transparency and credibility is a crucial step toward clinical translation.

As a promising deep learning model, GANs provide new possibilities for electrophysiological signal analysis. By effectively generating synthetic data samples, GAN is expected to achieve more outstanding achievements in electrophysiology and further promote the development and application of electrophysiological signal analysis.

## 6. Conclusions

In this overview, we explored the multiple applications of GANs in electrophysiological signal processing, including signal synthesis, classification, denoising, anomaly detection, modality translation and fusion, BCI application, de-identification, feature extraction and signal reconstruction. These applications not only enrich the technologies of electrophysiological signal processing but also demonstrate the potential of GANs as a powerful tool in signal processing. By learning the probability distribution and characteristics of signal data, GANs can provide new insights and solutions for the research, diagnosis, and clinical practice of electrophysiological signals, opening up broad possibilities for future research.

Despite the challenges associated with training stability, the adequacy of evaluation methods, and model interpretability, the role of GANs in electrophysiology will continue to expand. Continuous architectural innovations, coupled with the synergistic integration of multidisciplinary knowledge and the maturation of evaluation paradigms, will likely solidify the importance of GANs in both research and clinical settings. Through the discussion in this article, we hope to provide new perspectives and inspirations for researchers in medical image analysis and promote the application of GAN in electrophysiological signal analysis to achieve more breakthroughs.

## Figures and Tables

**Table 1:** Loss function variants formulas.

Study	Variants	Loss Formula
[42]	WGAN	$W(P_{r},P_{g})=\underset{\gamma \sim \Pi (P_{r},P_{g})}{\inf}E_{(x,y)\sim \gamma }[\|x-y\|],\;L=E_{x\sim P_{r}}[f_{w}(x)]-E_{x\sim P_{g}}[f_{w}(x)]$
[43]	BGAN	$\min_{B}\max_{D}E_{(s,r)\sim P_{\left(S,R\right)}}[\log D(r|s)+\log(1-D(B(r|s)))]$
[44]	EBGAN	$\nabla_{\theta }D_{KL}(q_{\theta }\|\tilde{p})=-E_{h(z)}\left[\sum_{m}\left(F_{\phi }(x)-b\right)\nabla_{\theta }\log g_{\theta }(x^{\left(m\right)}|z)\right]$
[45]	LSGAN	$\min_{\theta }S(\theta ,\phi^{*})=E_{x\sim p_{\mathrm{data}}(x)}L_{\theta }(x)+\lambda E_{x\sim p_{\mathrm{data}}(x),z\sim p_{z}(z)}{\left(L_{\theta }(x)-L_{\theta }(G_{\phi^{*}}(z))+△(x,G_{\phi^{*}}(z))\right)}_{+}$

**Table 2:** Generation of ECG based on GANs.

Study	Dataset	Architecture	Result Evaluation
[60]	MIT-BIH	GAN	Intuitive evaluation by loss function
[49]	MIT-BIH	BiLSTM-CNN	PRD: 51.799, RMSE: 0.215, FD: 0.803
[61]	MIT-BIH	WaveNet, SpectroGAN, WaveletGAN	WaveletGAN: GAN-train 89.07%, GAN-test 92.33% SpectroGAN: GAN-train 68.80%, GAN-test 99.96%
[63]	MIT-BIH	ME-GAN	AUC: 0.902
[65]	PTB-XL	GAN	All Plane: FD: 6.701, MSE: 0.017
[64]	PTB	GAN	SSIM: 0.92, PRD: 0.0721
[62]	PTB-XL, CCDD, CSE, Chapman	Two-dimensional BiLSTM GAN	Synthetic ECGs show diversity and variability similar to real ECGs.
[66]	MIT-BIH	BiLSTM-DC GAN, WGAN	BiLSTM-DC GAN: DTW 4.535, Fret 0.625, Euc 8.557 WGAN: DTW 4.401, Fret 0.681, Euc 8.486
[67]	MIT-BIH	CWGAN, CVAE	Fidelity: 77.35% Diversity: 96.81% Param: 108

**Table 3:** Generation of EEG based on GANs.

Study	Dataset	Architecture	Result Evaluation
[68]	Sleep-EDF	EEGGAN, SIGGAN	IS: 2.02, FID: 39.53
[29]	Self-Collection	Wasserstein GAN, EEG	IS: 1.281, FID: 120.854, EDmin: 0.034, SWD: 0.309
[69]	SEED	Wasserstein GAN (MG-CWGAN)	Normalized feature space within-group MMD: 0.026 ± 0.013

**Table 4:** Classification of ECG based on GANs.

Study	Task Type	Dataset	Architecture	Result Evaluation
[83]	Three-dimensional MHD Distortion Synthesis	INCART Getemed Schiller Siemens	GAN	Pre: 93.06%, Recall: 98.31%, F1: 95.61
[32]	GAN Effectiveness Evaluation	MIT-BIH	CNN, GAN	Acc: 97.69%, MCC: 92.59% Spe: 90.91%, Pre: 97.49% Recall: 95.13%, F1: 96.25%
[81]	CVD Classification	MIT-BIH-AR, CCDD	DB-GAN, Transformer, LSTM	Sen: 85.10%. Spe: 99.57% Predictive value: 75.32% Acc: 99.34%
[82]	Arrhythmia Subtype Classification	PTB-XL PhysioNet	ResNet, GAN	Pre: 82%, Recall: 80%, F1: 81%
[4]	Arrhythmia Signal Generation	MIT-BIH	LSTM, GAN	Acc: 98% Recall: 99% (N), 83% (AA), 99% (LBBB), 99% (RBBB)
[84]	ECG Generation (VAE)	MIT-BIH	ACE-GAN (GAN with auxiliary classifier)	Acc: 99% (SVEB), 99% (VEB) Sen: 87% (SVEB), 93% (VEB) Spe: 99% (SVE), 99% (VEB) PPRate: 85% (SVEB), 94% (VEB) F1: 86% (SVEB), 93% (VEB)
[85]	Arrhythmia Classification	MIT-BIH	GAN, CNN	Pre: 90.00% Sen: 99.77% Spe: 99.23% Acc: 98.30%
[80]	Dyskinesia Classification	MIT-BIH	TCGAN (Transformer, CNN)	Precision: 62.19% Recall: 62.96% F1 Score: 62.31% Accuracy: 97.29%
[86]	Class Imbalance Handling (ECG Generation)	PhysioNet, MIT-BIH	GAN-LSTM	Acc: 99.4% F1: 99.3% AUC: 99.5%
[79]	ECG Classification (Ensemble)	MIT-BTH, Atrial Fibrillation Detection, PTB Diagnostic	HeartNet (GAN, CNN)	Accuracy: 99.67% MCC: 89.24%
[87]	Noise ECG Generation	MIT-BTH	ProEGAN-MS, CNN	Acc: 98.55% Sen: 99.36% Pre: 92.89%
[88]	ECG Time Series Generation	MIT-BTH	LSTM, GRU, LSTM-LN, GAN	Acc: 95.2% Sen: 90.00% Spe: 100.00%

**Table 5:** Classification of EEG signals based on GANs.

Study	Task Type	Dataset	Architecture	Result Evaluation
[95]	Emotion Recognition	DEAP, SEED	GCNN, LSTM, GAN	Valence Acc: 94.87% Arousal Acc: 94.42% Dominance Acc: 94.78%
[96]	Emotion Recognition	DEAP, MAHNOB-HCI	CL, GAN, GNN	Valence Acc: 57.50% Arousal Acc: 70.00% Average Acc: 63.75%
[92]	Driver Fatigue Detection	Self-Collection	GAN, GCN	Acc: 96.25% Sen: 96.40% Spe: 96.20% Pre: 95.75%
[90]	Emotion Recognition	DEAP	GAN, MSSFAN, RSCN, SSLFTN	Valence Acc: 96.99% Arousal Acc: 97.15%
[93]	Sleep Stage Classification	CCSHS, Sleep-EDF, Sleep-EDF-V1	GAN, CNN	Acc: 86.10% Recall: 45.97%
[91]	RSVP Task	Self-Collection	GDANN, GAN	Acc: 91.63% Pre: 91.61% F1: 91.62% Recall: 91.76%
[50]	Emotion Recognition	RSVP	BWGAN-GP	AUC: 91.11%
[97]	Emotion Recognition	SEED, SEED-IV	VAE-D2GAN	IS: 2.206 FID: 11.016 MMD: 0.229
[98]	Artificial Sample Generation	Figshare	GAN, VAE, CNN	Validation Accuracy: 99.8% Validation Loss: 0.029
[89]	Emotion Recognition	SEED	VAE-GAN	Classification Acc: 97.21% (±1.04%)
[51]	Emotion Recognition	CASIA database	CS-GAN, CNN-LSTM	improvement On EEGs: +9.31%. improvement On real ERPs: +43.59%.
[99]	Emotion Recognition	Self-Collection	GAN, DNN, DEEG	acc: 0.984.
[94]	Sleep Scoring Classification	Self-Collection	SAGAN, CNN	F1: 95.7% ± 4.4% Acc: 97.1% ± 2.3%

**Table 6:** Generation of EMG and EOG signals based on GANs.

Study	Task Type	Modality	Dataset	Architecture	Result Evaluation
[52]	EMG Data Augmentation	EMG	sEMG signals	DCGAN	Fake vs. Real: 21.0% Mixed vs. Real: 13.4%
[103]	Sleepiness Detection	EOG; EEG	Self-collected	CWGAN, LSTM	Acc: 98.14% ± 0.75%
[101]	Data Augmentation	EMG	Self-collected	DCGAN	Acc: 93.5%
[102]	PD EMG Augmentation	EMG	Self-collected	DCGAN	DiscLoss: 0.004439, DTW: 98.532786 FFT MSE: 13.531477
[100]	sEMG Feature Generation	sEMG	Self-collected	EBGAN	Acc increase: 3.5%

**Table 8:** Electrophysiological anomaly detection with GANs.

Study	Task Type	Dataset	Architecture	Result Evaluation
[123]	IEDs Detection	Self-collected	VAE-cGAN (conditional GAN, VAE)	Acc: 77%
[124]	Seizure Forecasting	CHB-MIT, EPILEPSIAE, Freiburg Hospital	GAN	AUC: 77.68%
[125]	Seizure Forecasting	CHB-MIT	GAN	sen: 93%, spe: 92.5%
[126]	Epileptic Detection	CHB-MIT	GAN, 1DCNN	Sen: 93.53%, Spe: 99.05%, G-mean: 96.15%
[122]	Epileptic Detection	CHB-MIT	DCWGAN	Acc: 78.0%, AUC: 0.704
[121]	Epileptic Detection	Self-collected	GAN (Gram matrix)	AUC: 0.9372, Sen: 96.3%
[133]	Sleep Apnea Detection	PhysioNet, Bracelet Wearable PPG	1D-ConReNet, GAN, 1D-FSCNN	Acc: 91.45%; Sen: 89.62%; Spe: 92.58%; pre: 88.24%; F1: 88.93%; AUC: 0.9713
[130]	Arrhythmia Detection	MIT-BIH	ECG-ADGAN (Bi-LSTM, 1D-CNN)	Acc: 99.5%, Pre: 96.9% Recall: 91.8%, F1: 94.3%, AUC: 0.959
[53]	PxAF Detection	Self-collected	Pulse2Pulse GAN	Acc: 99.0%
[134]	Anomalous Rhythm Detection	MIT-BIH; CMU Motion Capture	BeatGAN	AUC: 94.75%, Pre: 91.43%
[127]	Arrhythmia Detection	MIT-BIH	AnoGAN	AUC: 94.75%, F-measure: 0.9143
[135]	Arrhythmia Detection	MIT-BIH	ACGAN, LSTM	Acc: 99.81%, Sen: 99.53%, Spe: 99.88%
[5]	Arrhythmia Detection	MIT-BIH	MadeGAN (MemAE), 1D CNN	AUROC: 0.950, AUPRC: 0.922 Recall: 0.964, Pre: 0.967 F-score: 0.965, Acc: 0.967
[28]	Myocardial Infarction Detection	MIT-BIH	SLC-GAN	Acc: 99.06%, Recall: 99.33% Pre: 99.14%, Spe: 98.65%
[129]	Time Series ECG Anomaly Detection	ECG5000, MIT-BIH	VAE-GAN	AUC: 0.93, F1: 93.2%, Acc: 90.1% Recall: 95.1%, Pre: 91.4%

**Table 9:** Modal Conversion of electrophysiology with GANs.

Study	Modality	Dataset	Architecture	Result Evaluation
[141]	EEG to stimulating images	ImageNet	GAN (CapsNet, Transformer and ensemble strategies)	Mean Acc: 77.79% on Baseline model
[137]	ECG to PPG	VitalDB	GAN (LSTM, CNN)	PRD: 31.9% ± 10.3, CC: 0.949 ± 0.047
[56]	EEG to SIQR	Self-Collection	MF-GAN, BAF encoder, MST EEG encoder	Kappa: 88.617%, Hamming: 0.0763, Acc: 94.137%
[138]	PCG to ECG VCG to 12-lead ECG	PhysioNet	Cycle-GAN, GAN (U-Net)	Analyze consistency by qualitative result
[139]	sEMG to Speech waveform	Self-Collection	GAN (multi-scale discriminator, multi-period discriminator)	CER: 0.3243, TER: 0.2613, MCD: 8.45 dB Log F0 RMSE: 0.40, F0 CORR: 0.71, FO V/U: 0.80
[136]	EEG to fMRI	Self-Collection	BMT-GAN (Cycle-GAN and non-adversarial structure.)	MSE: 128.6233, PSNR: 27.0376, SSIM: 0.8627, VIF: 0.3575, IFC: 2.4794
[140]	EEG to Visual Saliency Maps	EEG-ImageNet	GDN-GAN (Chebyshev Graph Convolution)	Pre: 98.56%, F1: 98.56%, Recall: 98.56%

**Table 10:** BCI applications with GANs.

Study	Objective	Dataset	Architecture	Result Evaluation
[148]	EEG Synthesis by Category	Competition IV, self-collection	CVAE-GAN	IS: 1.357, FID: 11.364, SWD: 0.067
[145]	MI EEG Noise Generation	BCI IV 2a	GAN (With SGD)	ACC: 86.15%
[146]	Motor Imagery Classification	self-collection	CycleGAN, CNN	Average 7.99% boost in CNN, 1.34% in SVM
[147]	Emotion Recognition Calibration	DEAP, SEED-V, GraffitiVR	CWGAN-GP	acc: 93% on SEED-V, 99% on DEAP, 97% on GraffitiVR
[144]	Simulated-to-Real EEG Transfer	ME-BCI	CGAN	Acc: 86.5% on Subspace KNN

**Table 11:** Signal de-identification with GANs.

Study	Objective	Dataset	Architecture	Result Evaluation
[153]	ECG Synthesis for Privacy	GESUS, Inter99	WaveGAN (Pulse2Pulse)	Synthetic ECGs closely matched real ECGs in heart rate, QT, QRS, PR intervals, and waveform amplitude (heart rate: real 70 ± 8 bpm, synthetic 70 ± 7 bpm).
[149]	ECG De-identification	Self-Collection	GAN (With ODE Loss)	Arrhythmia detection accuracy: synthetic data achieved 96% (type N, val acc 0.89) and 85% (type S, val acc 0.99), consistent with real data in identity and disease detection.
[151]	Arrhythmia & ECG Authentication	MIT-BIH	GAN	Data augmentation increased CNN accuracy by 7.99% and SVM by 1.34%.
[152]	Epileptic EEG Synthesis	EPILEP-SIAE	EpilepsyGAN (CGAN)	Median recall of synthetic data: 3.2%, close to random, effectively reducing re-identification risk and enhancing privacy.
[36]	Synthetic Data Generation	KEEL	GAN	De-identified data generated; generation results not detailed.
[154]	EEG-based Human Identification	PhysioNet	ABCL-EHB (GAN, CNN-LSTM)	EEG identification F1-score: 99.65% (64 channels), 99.64% (14 channels), 99.55% (9 channels), with a 3.21% improvement at 9 channels.
[150]	EMG Style Transfer & Classification	CapgMyo	GAN, Transformer	Average confusion success: 99.41%; manipulation success rate: 91.51%.

**Table 12:** Electrophysiological feature extraction with GANs.

Study	Modality	Objective	Dataset	Architecture	Result Evaluation
[155]	EEG	EEG Feature Filtering	UCI EEG	CycleGAN	Alcoholism Characterization Removal Rate: 66.3%
[54]	ECG	FECG Extraction (STFT + GAN)	MIT-BIH ST change	GAN	PCDB: SE 92.37%, PPV 93.69%, F1 93.02%
[156]	ECG	Noncontact Respiratory Monitoring (cEDR)	cEDR	TF-GAN	Signal CC > 0.5: 86.3% RMSE: 0.96 ± 0.12 bpm Bland-Altman concordance: 94.83% ± 0.30

**Table 13:** Electrophysiological signal reconstruction with GANs.

Study	Modality	Objective	Dataset	Architecture	Result Evaluation
[157]	EEG	LSS-EEG Reconstruction	AO, GAL, MI	WGAN (With TSF-MSE)	AO: 67.67%, GAL: 73.89%, MI: 64.01%
[158]	BCG, ECG	ECG Reconstruction from BCG	Self-Collection	Att-SNGAN	MAE: 0.0651, RMSE: 0.1008, FD: 0.2356

**Table 14:** Maturity and clinical status of GANs across signal modalities.

Modality	Maturity Level	Data Availability	Typical Tasks	Leading Model	Major Open Gaps
ECG	Established	High (e.g., PTB-XL, MIT-BIH)	Synthesis, Anomaly Detection	WGAN-GP, CWGAN	Preserving P-QRS-T timing
EEG	Established	High (e.g., TUH, CHB-MIT)	Synthesis, Classification, Denoising	EEG-GAN, Transformer-GAN	Spatiotemporal consistency
EMG	Emerging	Moderate (Self-Collection)	Gesture Classification, Data Augmentation	DC-GAN, CGAN	Robustness to movement artifacts
EOG	Nascent	Low (Self-Collection)	Artifact Removal, Eye-movement Tracking	Basic GAN variants	High inter-device variability

## Data Availability

No new data were created or analyzed in this study.

## Abbreviations

The following abbreviations are used in this manuscript:

EEG	Electroencephalogram
ECG	Electrocardiogram
EMG	Electromyogram
EOG	Electrooculogram
GANs	Generative Adversarial Networks
BCI	Brain–Computer Interfaces
DL	Deep learning
PET	Positron emission tomography
MRI	Magnetic resonance imaging
CT	Computed tomography
G	Generator
D	Discriminator
DC-GANs	Deep Convolutional GANs
CNN	Convolutional Neural Networks
VAE	Variational autoencoder
MEG	Magnetoencephalography
WGANs	Wasserstein Generative Adversarial Networks
BGANs	Boundary-Seeking Generative Adversarial Networks
EBGANs	Energy-Based GANs
CGANs	Conditional GANs
ACGANs	Auxiliary Classifier GANs
SAGANs	Self-Attention GANs
FD	Frechet distance
Bi-LSTM	Bidirectional long short-term memory network
CWGAN	Conditional Wasserstein GAN
DB-GAN	Dual-branch GAN
DGGN	Domain Generation Graph Network
GCN	Graph convolution
PLI	Power Line Interference
BW	Baseline Wander
EM	Electrode Motion
MA	Muscle Artifacts
RN	Random Noise
SNR	Signal-to-noise ratio
BCG	Ballistocardiogram
sEMG	Surface electromyography
RRMSE	Relative root-mean-square error
ICA	Independent component analysis
scEEG	Scalp-mounted EEG
iEEG	Intracranial EEG
IEDs	Interictal epileptiform discharges
NSSI	Non-suicidal self-injury
SAS	Sleep Apnea Syndrome
PPG	Photoplethysmogram
PCG	Phonocardiogram
SIQR	Stereoscopic image quality ranking
GDN-GAN	Geometric deep network-based GAN
HATL	Heterogeneous adversarial transfer learning
ERD	Event-related desynchronization
ERS	Event-related synchronization
STFT	Short-time Fourier transform
FECG	Fetal electrocardiographic
TF-GAN	Time–Frequency Domain GAN
TSF-MSE	Time–Space–Frequency
Att-SNGAN	Attention-based Spectral Normalization GAN
HRV	Heart rate variability
XAI	Explainable Artificial Intelligence
FID	Fréchet Inception Distance
IS	Inception Score
RMSE	Root Mean Square Error
PRD	Percent Root-mean-square Difference
AUC-ROC	Area Under the Curve of the Receiver Operating Characteristic
AUPRC	Area Under the Precision–Recall Curve
FDR	False Discovery Rate
SSIM	Structural Similarity Index
PSNR	Peak Signal-to-Noise Ratio
MCD	Mel-Cepstral Distortion
CC	Pearson Correlation Coefficient
